# Monoclonal gammopathy of undetermined significance is associated with prostate cancer in a population-based cohort study

**DOI:** 10.1038/s41598-021-98803-1

**Published:** 2021-09-29

**Authors:** Nicola Hornung, Mirjam Frank, Nico Dragano, Jan Dürig, Ulrich Dührsen, Susanne Moebus, Raimund Erbel, Andreas Stang, Karl-Heinz Jöckel, Börge Schmidt

**Affiliations:** 1 Institute for Medical Informatics, Biometry and Epidemiology, University Hospital of Essen, University of Duisburg-Essen, Hufelandstraße 55, 45122 Essen, Germany; 2 Department of Medical Sociology, University Clinic Düsseldorf, Düsseldorf, Germany; 3 Department of Hematology, University Hospital Essen, University of Duisburg-Essen, Essen, Germany

**Keywords:** Cancer, Haematological diseases, Epidemiology

## Abstract

Register-based studies indicate a possible association of monoclonal gammopathy of undetermined significance (MGUS) and prostate cancer (PCa). Aim of the present study was to investigate the relationship between MGUS and PCa considering potentially shared risk factors. Data from the prospective population-based Heinz Nixdorf Recall cohort study of 2.385 men (age 45–85) were analyzed. MGUS was determined at three points in time; cases of cancer were assessed annually. Potentially shared risk factors were assessed at baseline. Hazard ratios (HR), adjusted for age and educational attainment, and corresponding 95%-confidence intervals (95%-CI) were calculated. 157 cases of MGUS and 143 incident cases of PCa were detected. Of 19 participants diagnosed with both, MGUS and incident PCa, only in one case MGUS did not clearly occur before PCa. MGUS was associated with PCa presenting a HR of 2.00 (95%-CI: 1.23–3.25). Stratified by isotype, IgM-MGUS showed the strongest association with PCa. There was no relevant change of the effect estimate when adjusting for potentially shared risk factors. We were able to give supporting evidence for an association between MGUS and PCa and pointed out its temporality. There was no indication that the observed association is due to shared risk factors. The present study indicated that different isotypes of MGUS differ in the strength of the effect on PCa-risk. Based on these findings, future studies investigating the pathophysiological background of the association will be needed.

## Introduction

Monoclonal gammopathy of undetermined significance (MGUS) is a well-known precursor of lymphoid malignancies^1^. The prevalence of MGUS in populations aged ≥ 50 years has been estimated at 3–4% with a risk of progression to multiple myeloma of ~ 1% per year^1^. Some studies have also indicated an association between MGUS and risk of different types of solid cancers such as prostate cancer (PCa). Confounding due to shared genetic susceptibility and common risk factors has been considered a possible explanation^2–4^. Some physicians have also reported associations between MGUS and PCa in case reports^5,6^.

The origin of MGUS is largely unknown, but familial accumulations, which have been observed in some studies, imply a genetic influence^2^. Risk factors discussed for MGUS include a high body-mass-index (BMI), black skin color, male sex, higher socio-economic status, certain genetic polymorphisms and higher age^7–9^.

PCa is one of the most frequent malignant diseases among men^10^. Metastases are predominantly found in the iliac lymph node stations (lymphogenic) and in the skeleton (haematogenic)^11^. The main risk factor for PCa is age^12^. Other factors discussed are dietary factors (high intake of calcium, e.g. through frequent consumption of dairy products or meat), vitamin D deficiency, black skin colour and familial history (i.e., genetic factors)^12,13^.

To our knowledge the association of MGUS and PCa has not yet been examined in population-based cohort studies. Based on the previous evidence^2–6^, the aim of the present study was to investigate the association of MGUS and PCa including the temporality between MGUS and PCa diagnosis as well as potentially shared risk factors that may explain the association. In addition, the impact of MGUS isotypes on the association of MGUS with PCa was explored as well as the association of PCa and progression of MGUS to multiple myeloma. Investigating the association between MGUS and PCa and identifying shared risk factors may help to better understand disease etiology and inform prevention strategies.

## Methods

### Study population

The Heinz Nixdorf Recall Study is a prospective population-based cohort study from the Ruhr area in Germany. Participants were recruited between 2000 and 2003 using a random sample of the resident population of the cities Essen, Bochum and Mülheim/Ruhr. The baseline recruitment efficacy proportion was 56%. Participants were invited for a 5- and 10-year follow-up examination^14^. In total 4814 participants aged 45–75 years gave informed consent. The study was approved by the institutional ethics committee of the University Duisburg-Essen and was conducted according to the guidelines and recommendations for ensuring Good Epidemiological Practice (https://www.dgepi.de/assets/Leitlinien-und-Empfehlungen/Recommendations-for-good-Epidemiologic-Practice.pdf). The study design has been described in more detail by Schmermund et al.^15^.

### Prostate cancer (PCa)

PCa diagnosis was assessed by a computer-assisted personal interview (CAPI) at baseline examination and annually during follow-up by a self-administered questionnaire^16^. Participants were asked about hospital stays, operations or if they had ever been diagnosed with cancer (and if so, what type of cancer). CAPI was repeated at the 5-year and 10-year follow-up examination to check and supplement information from the annually questionnaires. If participants could not take part in the follow-up investigations, standardized telephone interviews with the participants or their relatives were conducted. Drop-outs received a non-responder questionnaire and death certificates were requested for deceased participants in order to determine the cause of death. For all reported cancer diagnoses, operations or hospital stays, medical records were requested. Finally, validated cases of cancer were entered into a database with relevant data like ICD-10-code (i. e., C61 for PCa), localization, staging and date of diagnosis.

### Monoclonal gammopathy of undetermined significance (MGUS)

Serum blood samples of study participants used for MGUS screening were collected at baseline, 5-year and 10-year follow-up examination and stored at −80 °C^17^. In order to detect MGUS, standard serum electrophorese and screening immunofixation using pentavalent antisera were performed. In case of a visible or suspected monoclonal band an immunofixation using antisera against γ, α, µ, κ and λ was carried out. Nephelometry was used to determine free light chains and the κ/λ-ratio was calculated. Results were classified according to reference ranges published by Katzmann et al.: κ: 3.3–19.4 mg/l; λ: 5.7–26.3 mg/l; κ/λ: 0.26–1.65^18^. Overall, diagnostic procedures included laboratory results, past medical history and serum concentration of the m-protein. MGUS was diagnosed in accordance to the criteria of the International Myeloma Working Group 2003: serum m-protein concentration < 30 g/l, < 10% bone marrow clonal plasma cells and absence of end-organ or tissue damage caused by MGUS^19^. The occurrence of multiple myeloma was assessed using the same validation procedure as for PCa.

### Potential risk factors

Next to the confounders age and educational attainment (as indicator of socio-economic status), discussed risk factors for both, MGUS and PCa, were selected by reviewing the literature^9,20–32^ and were included in the analysis as potential underlying causes (i. e., confounders) for an association between MGUS and PCa. Information assessed at baseline was available for body mass index (BMI), physical activity, smoking, alcohol consumption, blood serum cholesterol, intake of statins, diabetes mellitus and dietary factors. Educational attainment was assessed by standardized face-to-face interviews, coded according to the International Standard Classification of Education as total years of formal education by combining school and vocational training and then divided into four categories (≤ 10 years, 11–13 years, 14–17 years, ≥ 18 years)^33^. BMI (kg/m^2^) was calculated using measured body height and weight. Physical activity (metabolic-equivalent-task-hours/week) was assessed as part of the CAPI. Smoking was categorized into: current smoker (smoking cigarettes during the past year), former smoker (smoking cigarettes before the past year) and never-smoker. Alcohol consumption (g/week) was computed from reported number and type of drinks per day. Diabetes mellitus was defined as either of the following criteria: reported history of diabetes mellitus, intake of glucose lowering drugs, measured fasting blood glucose level > 125 mg/dl or non-fasting blood glucose level > 200 mg/dl or measured HbA1c > 6.5%^34^. Total cholesterol, LDL-Cholesterol and HDL-Cholesterol (mg/dl) were determined from blood serum samples taken at baseline examination. Information on intake of statins was assessed using a standardized assessment of medication history of the last 7 days at baseline examination. Information on dietary intake of dairy products, fish, fruits, raw and cooked vegetables assessed using a validated food frequency questionnaire was dichotomized in high vs. low consumption^35^. High consumption was defined as an intake of at least 4–6 times/week for dairy products, fruits and vegetables and at least 1–3 times/week for fish. In order to reflect possible genetic influences, the family history of PCa was taken into account. Family history of PCa was assessed at baseline and included PCa diagnoses of the participant’s father and up to nine brothers.

### Statistical analysis

Data of 2385 male participants with non-missing information on MGUS were included in the analyses (Figure S-1). Descriptive analysis was carried out for the whole analysis population as well as stratified by MGUS status and/or PCa. For each participant having MGUS as well as PCa (N = 21), the examination date of first MGUS detection and the date of PCa diagnosis were plotted in order to determine the temporality between MGUS and PCa. Prevalent cases of PCa at baseline (N = 39) were then excluded from further analyses. Hazard ratios (HR) with corresponding 95%-confidence intervals (95%-CI) were calculated by using Cox proportional hazards regression with MGUS status (cumulating prevalent and incident MGUS) as independent and incident PCa as dependent variable. Validity of the proportional hazards assumption was confirmed prior to analysis and assessed by visual inspection of Schoenfeld residuals. The median observation time was 13.6 years (inter quartile range 9.6–15.5 years) starting at baseline.

First, to quantify the strength of the association between MGUS and PCa a basic model (BM) was fitted under the assumption that none of the discussed risk factors acted as confounder for the association between MGUS and incident PCa. Thus, the minimal adjustment set included only age and education.

Second, to assess any confounding by potential risk factors the BM was then additionally adjusted for each potential risk factor separately to quantify the risk factors impact on the HR obtained for the association between MGUS and incident PCa. Finally, a full model (FM) was fitted under the assumption that all discussed risk factors acted as confounders for the association between MGUS and PCa. The minimal adjustment set under this assumption included all discussed risk factors (except total cholesterol due to its strong association to LDL). Participants with missing information on potential risk factors and participants with missing information on education (N = 10) were only excluded from the respective regression models. Complete case (N = 1938) sensitivity analysis was performed to check for the influence of missing data on the study results by excluding all participants with any missing data.

Third, the BM was also calculated for MGUS stratified by isotype to investigate whether the strength of the observed association varied between different isotypes.

As not every MGUS case included in the analysis was prevalent at study baseline, the BM was also calculated with an observation time starting at the actual examination date of first MGUS detection for each incident MGUS case (i.e., either at the 5- or 10-year follow-up examination). This led to a reduced median time to event of 13.4 years (inter quartile range 9.3–15.5 years). In addition, to determine the association of the potential risk factors with MGUS, age- and education-adjusted logistic regression models were fitted to estimate odds ratios (OR) and 95%-CI, while the association of potential risk factors with PCa was analyzed using age- and education-adjusted Cox proportional hazards regression models. All analyses were carried out using IBM SPSS Statistics 24. For original data, please contact the corresponding author.

## Results

The characteristics of the study cohort are presented in Table 1. The mean age was approximately 60 years. 47.1% of the participants had at least 14 years of formal education. Overall, there were 157 cases of MGUS and 182 cases of PCa. The isotype IgG was found in 58.6% of all MGUS cases (Table 2). IgA-MGUS was detected in 14.7% and IgM in 19.8%. In 7.0% the subtype remained unknown. Eleven participants were diagnosed with incident multiple myeloma. There were 143 incident cases of PCa, showing an incidence rate of 4.97 (95%-CI: 4.22–5.86) per 1000 person years. The mean age at PCa diagnosis was 66.7 (± 6.8) years. The mean age at first MGUS detection was 64.7 (± 8.1) years.

**Table 1:** Characteristics of the male study participants of the Heinz Nixdorf Recall (HNR) study.

N^b^	2385 (100.0%)
Age^a^ years	59.7 (± 7.8)
**Education**^**b**^ [10]	
≤ 10 years	120 (5.1%)
11–13 years	1,135 (47.8%)
14–17 years	796 (33.5%)
≥ 18 years	324 (13.6%)
**PCa**^**b**^	
Prevalent at baseline	39 (1.64%)
Incident	143 (6.00%)
**MGUS**^**b**^	157 (6.58%)
Prevalent at baseline	98 (4.1%)
Incident	59 (2.5%)
Multiple myeloma^b^ (incident)	11 (0.46%)
Body mass index (kg/m^2^)^a^ [12]	28.2 (± 4.0)
Physical activity (MET-hours/week)^c^ [39]	35.0 (15.7–66.0)
Smoking^b^ [5]	
Never smoker	665 (27.9%)
Past smoker	1,104 (46.4%)
Current smoker	611 (25.7%)
Alcohol (g/week)^c^ [42]	46.3 (6.9–118.6)
Diabetes mellitus^b^	473 (19.8%)
Total cholesterol (mg/dl)^a^ [8]	224.7 (± 38.3)
LDL-cholesterol (mg/dl)^a^ [16]	145.1 (± 35.5)
HDL-cholesterol (mg/dl)^a^ [10]	51.0 (± 14.4)
Statin intake^b^ [171]	289 (13.1%)
High milk consumption^b^ [126]	655 (29.0%)
High yoghurt/quark consumption^b^ [104]	983 (43.1%)
High cheese consumption^b^ [84]	1,140 (49.5%)
High fruits consumption^b^ [41]	1,428 (60.9%)
**Highvegetableconsumption**^b^	
Raw [42]	588 (25.1%)
Cooked [40]	705 (30.1%)
High fish consumption^b^ [43]	820 (35.0%)
PCa in first degree relevants (family history)	69 (2.9%)

*MGUS* monoclonal gammopathy of undetermined significance, *PCa* prostate cancer.

^a^Mean (± standard deviation).

^b^Number (%).

^c^Median (inter quartile range), [number of missing values].

**Table 2:** Characterization of MGUS cases (N = 157).

**MGUScases**^**b**^	
Type IgG Type IgA Type IgM Others	92 (58.6%) 23 (14.7%) 31 (19.8%) 11 (7.0%)
M-protein (g/dl)^c^ Not detectable^b^	5.20 (3.80–2.50) 70 (44.6%)
Pathologic FLC-ratio^b^ [5]	32 (21.1%)

*MGUS* monoclonal gammopathy of undetermined significance.

^b^Number (%).

^c^Median (inter quartile range).

**Figure 1:**
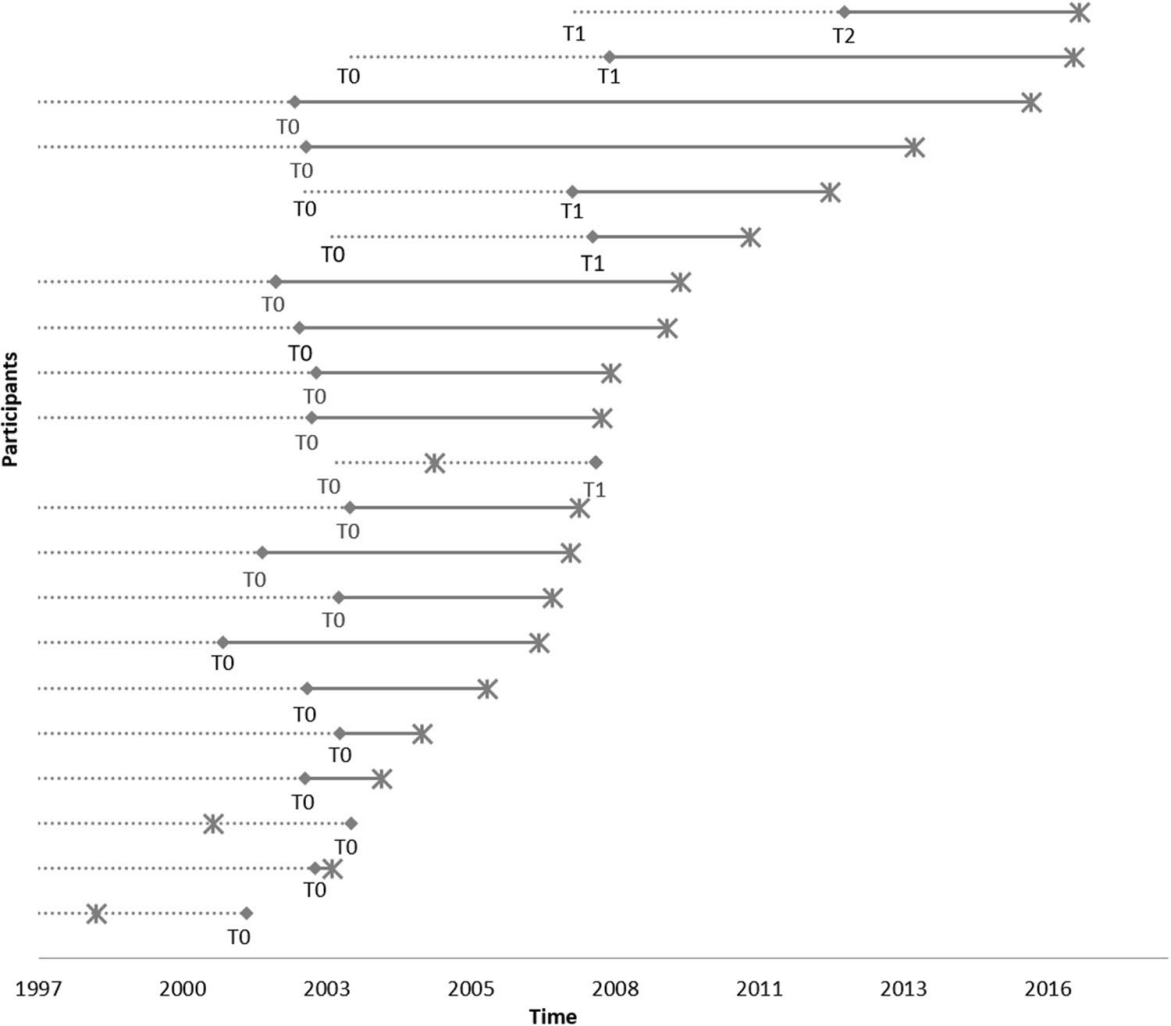
Timeline of MGUS detection and prostate cancer diganosis for study participants with both diagnoses (N = 21). Asterisk: date of PCa diagnosis; diamond: examination visit of MGUS detection; dotted line: time interval of possible MGUS occurrence before MGUS detection; T_0_ = date of baseline examination, T_1_ = date of 5-year follow-up examination, T_2_ = date of 10-year follow-up examination.

Twenty-one participants were diagnosed with both, MGUS and PCa. None of them developed multiple myeloma. The temporality between MGUS detection and PCa for each of those participants is shown in Fig. 1. In 18 of the 21 participants, MGUS was detected before PCa was diagnosed (Fig. 1). In the remaining 3/21 cases (including 2/21 prevalent PCa cases that were excluded from further analysis), PCa was diagnosed in the time interval before the examination date of first MGUS detection where MGUS may have been already occurred but not detected.

The characteristics of the study cohort stratified by MGUS status or incident PCa did not reveal strong differences in the distribution of risk factors (Table S-1). Age was slightly lower in participants without MGUS and PCa, while in the group with PCa and the group without MGUS and PCa a lower proportion of participants in the highest education group was observed.

**Figure 2:**
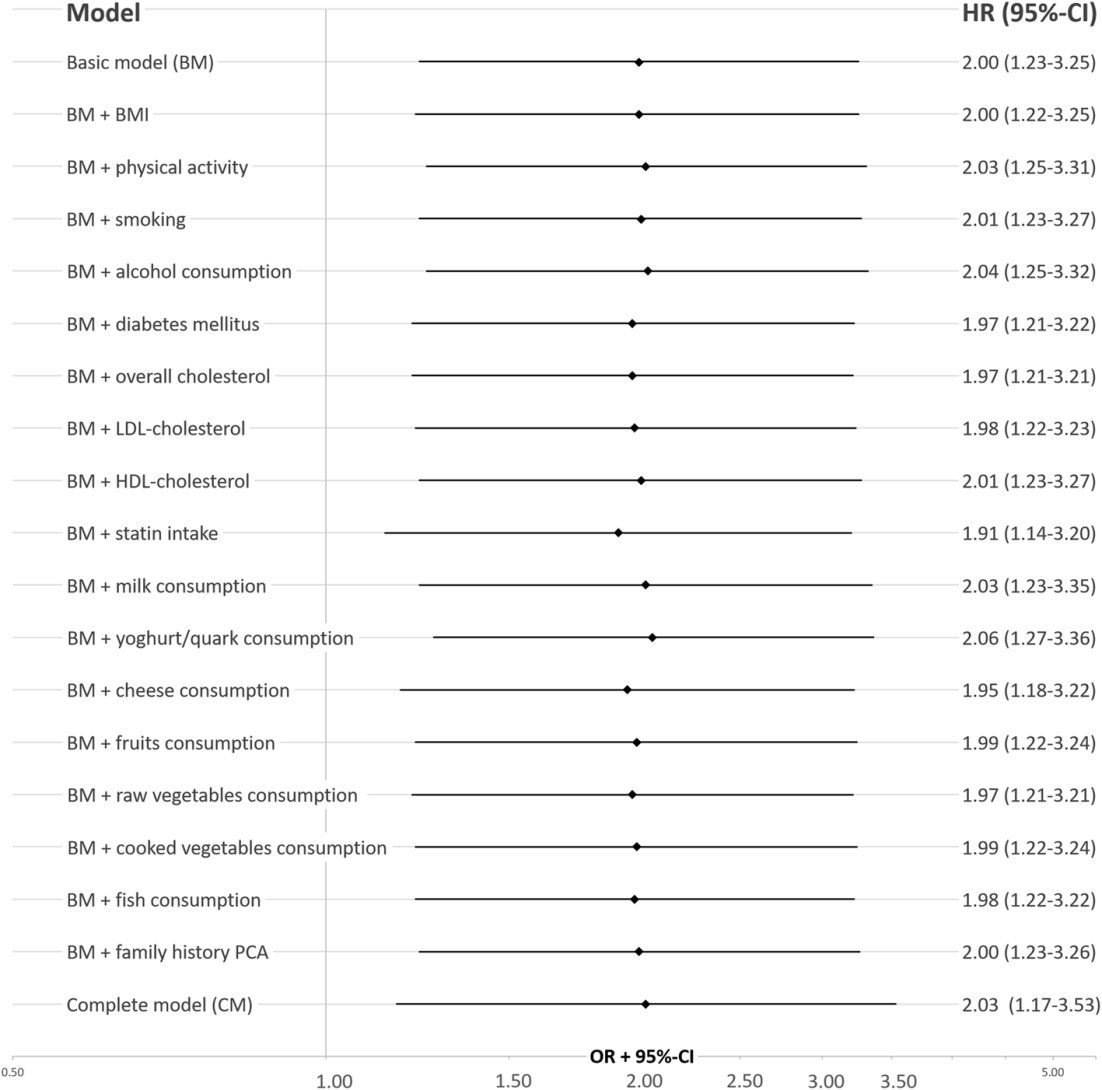
Hazard ratios (HR) and corresponding 95%-confidence intervals (95%-CI) for the association of monoclonal gammopathy of undetermined significance (MGUS) and incident prostate cancer (PCa), subsequently adjusted for potential risk factors. *BM* basic model (i.e., adjusted for age and education), *[N]* number of observations included in the model, *BMI *body mass index, *LDL* low density lipoprotein, *HDL* high density lipoprotein, full model = adjusted for age, education and all potential risk factors (except total cholesterol).

MGUS was associated with PCa in the age- and education-adjusted BM showing a hazard ratio for the effect of ever having MGUS (HR_MGUS_) on incident PCa of 2.00 (95%-CI: 1.23–3.25). HR_MGUS_ changed only slightly when potential risk factors were included in the regression model (Fig. 2). The strongest reduction of the effect size estimate was observed when adjusting for statin intake (HR_MGUS_ 1.91, 95%-CI: 1.14–3.20). The full model including all risk factors still showed a HR_MGUS_ of 2.03 (95%-CI: 1.17–3.53). The complete case sensitivity analysis showed similar results (Table S-2). However, there was no reduction of the HR_MGUS_ estimate when adjusting for statin intake, but a small change of HR_MGUS_ when adjusting for total cholesterol (Table S-2).

Except for the confounders age and education, none of the potential risk factors included in the analysis showed strong indication for an association with MGUS (Table S-3). Age was also associated with PCa, while diabetes mellitus (HR 0.53; 95%-CI: 0.32–0.88) was indicated as a protective factor for incident PCa (Table S-4).

**Figure 3:**
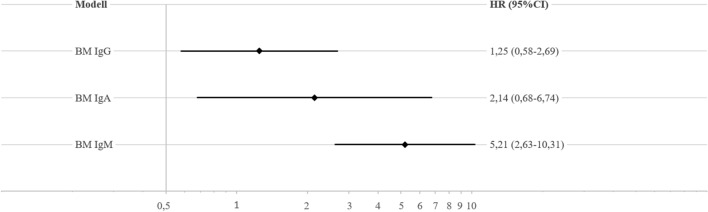
Hazard ratios (HR) and corresponding 95%-confidence intervals (95%-CI) for the association between individual isotypes of monoclonal gammopathy of undetermined significance (MGUS) and incident prostate cancer (PCa) adjusted for age and education using the group with no MGUS as reference (N = 2336). *Ig* immunoglobulin.

MGUS categorized by isotype showed an association of each isotype with PCa using the group with no MGUS as reference (Fig. 3). The strongest effect was observed for IgM-MGUS with a HR of 5.21 (95%-CI: 2.63–10.31), followed by IgA-MGUS with a HR of 2.14 (95%-CI: 0.68–6.74). IgG-MGUS showed the weakest association presenting a HR of 1.25 (95%-CI: 0.58–2.69). However, effect size estimates were less precise for single isotypes due to the low number of PCa cases per isotype stratum.

## Discussion

Aim of the present study was to investigate the association between MGUS and PCa and to examine the impact of potentially shared risk factors. While the study results showed an association of MGUS and PCa, no indication of shared lifestyle risk factors was observed. Moreover, the data of this study population enabled an investigation of the temporal sequence. Results indicated that MGUS seems to occur before PCa diagnosis. With regard to the isotypes, the analyses showed that IgM-MGUS may have the strongest association with PCa.

An increased risk of PCa in multiple myeloma or MGUS patients and their relatives has previously been suggested mainly by results of hospital- and register-based studies^2–4,36–38^. Most authors have suspected shared risk factors or shared genetic susceptibility as reason for this association^2–4,36,37^. Our results, however, gave no supporting evidence that the risk factors under investigation are operating as shared cause of the association, as there was no substantial change in the effect of MGUS on PCa when taking risk factors into account. The consideration of the family history concerning PCa in our study also gave no indication that strong genetic susceptibility seems to play a role as underlying cause for the association between MGUS and PCa.

The stronger association of IgM-MGUS in our study gave indication that there may be high and low risk isotypes of MGUS concerning development of PCa. The IgM-MGUS isotype, however, is usually characterized by a higher rate of progression into Waldenström’s Macroglobulinemia instead of MM^39^. Previous studies have already suggested a possible association of Waldenström’s Macroglobulinemia and PCa^40^. The results of the present study indicate that those previous findings might result from the association of Waldenström’s Macroglobulinemia’s precursor disease IgM-MGUS with PCa.

Kao et al. have analyzed the pathogenesis of multiple myeloma and PCa and developed a model, in which cytokines (such as IL-6, IGF-1 and VEGF) and immune suppression caused by multiple myeloma are responsible for an increased progression of prostatic intraepithelial neoplasia as a risk factor to detectable stages of PCa^4^. Our results may allow an extension of this model since we showed an association of PCa with MGUS, the precursor state of multiple myeloma. Cytokines and immune deficiency can still be considered in this extension, as the secretion of IL-6, which supports initiation and progress of PCa, has been increased in subjects with MGUS in previous studies^41,42^. IGF-1 might accelerate the risk of PCa, but has been suspected to prevent progression from MGUS to multiple myeloma^41,43^. This could explain why none of the participants with MGUS and PCa had multiple myeloma. Immune deficiency still has to be taken into account since 29–38% of the subjects with MGUS have presented depressed polyclonal immune globulins in a study by Kyle et al.^44^. However, further research is needed to investigate these hypothesized pathophysiological mechanisms that may explain the observed association.

Previous studies have not been able to give information on the temporality between MGUS and PCa diagnosis, since subjects with prevalent MGUS or multiple myeloma and sometimes their relatives have been included and analyzed concerning different types of cancer^2,3,36–38^. The design of the Heinz Nixdorf Recall study had the strength of being a prospective cohort study with population-based MGUS-screening at different points in time, which is superior to hospital- or register-based studies. Our results indicated that MGUS seems to occur before PCa diagnosis.

Next to the prospective and population-based design of our study, the use of a sensitive MGUS screening method, a long time period of follow up and extensive validation to confirm the cases of PCa represented further strengths. However, one may argue that data on PCa was based on clinical diagnosis and therefore might underestimate the number of actual cases. Due to screening-recommendations for PCa in Germany we assumed the number of undetected cases were negligible. Surveillance bias concerning the diagnosis of PCa in participants with MGUS is very unlikely an explanation of the observed association between MGUS and PCa, since participants were not informed if MGUS was detected. This approach was approved by the local ethical committee and in accordance with former clinical recommendations for the management of MGUS. A limitation was that urine electrophoresis, advanced imaging and bone marrow biopsies were not carried out for MGUS diagnosis. This might have led to a small number of smoldering MM or MM being misclassified as MGUS. Light-chain MGUS cases were not included in the statistical analysis since the number of participants fulfilling the criteria of light-chain MGUS (N = 22) was too low to expect meaningful results. Ethnicity as a risk factor could not be investigated due to the ethnically homogeneity of our cohort. Also, PCa was not categorized by aggressiveness. Future studies will be needed to determine whether MGUS is associated with both, aggressive and indolent PCa.

The present study gave supporting evidence for an association between MGUS and PCa using data from a population-based cohort study. We were able to point out the temporality of MGUS detection and PCa diagnosis and observed some indication for different effects between MGUS isotypes. These findings can be important for future studies investigating the pathophysiological background of the association in order to determine potential pathways from MGUS to PCa. Since PCa is the most frequent cancer in German men and MGUS presents a prevalence of about 3–4%, understanding the nature of this association can be important for clinical decision-making^1,17^.

## Supplementary Information

Supplementary Information.

## Data Availability

The datasets used and analyzed during the current study are available from the corresponding author on reasonable request.
