# Supplementary material for: Monoclonal gammopathy of undetermined significance is associated with prostate cancer in a population-based cohort study
Source: Sci Rep. 2021 Sep 29;11:19266. doi: 10.1038/s41598-021-98803-1 (PMC8481402; doi:10.1038/s41598-021-98803-1)
Supplement: Supplementary file 1 — Supplementary Information. [file 41598_2021_98803_MOESM1_ESM.pdf]

Supplement

Figure S-1: Flow chart: selection of analysis populations.

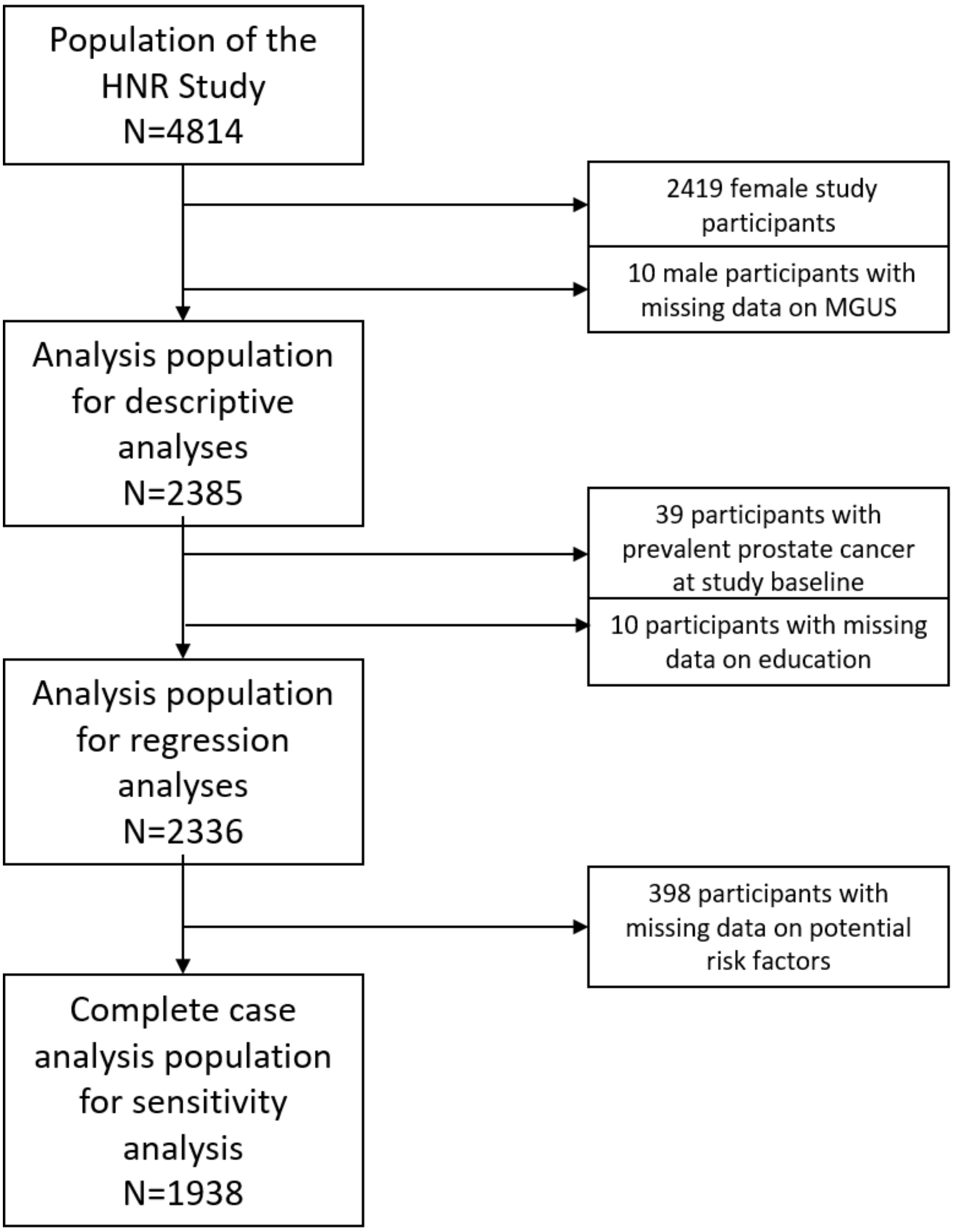

Table S-1: Characteristics of the male study participants of the Heinz Nixdorf Recall (HNR) Study excluding cases of prostate cancer (PCa) prevalent at study baseline, stratified by monoclonal gammopathy of undetermined significance (MGUS) and/or PCa. (N=2,346).

	**No MGUS or PCa**	**MGUS and PCa**	**Only MGUS**	**Only PCa**
**N** ‡	2,058 (86.7%)	21 (0.9%)	136 (5.7%)	161 (6.8%)
**Age***	59.3 (±7.8)	63.3 (±6.1)	61.7 (±7.6)	62.7 (±7.0)
**Education** ‡ [10]				
≤ 10 years	108 (5.2%)	1 (4.8%)	4 (3.0%)	7 (4.3%)
11-13 years	995 (48.3%)	9 (42.9%)	56 (41.5%)	75 (46.6%)
14-17 years	677 (32.9%)	7 (33.3%)	50 (37.0%)	62(38.5%)
≥ 18 years	278 (13.5%)	4 (19.0%)	25 (18.5%)	17 (10.6%)
**Body mass index** (kg/m^2)* [12]	28.1 (±4.0)	27.9 (±3.3)	28.9 (±4.3)	28.5 (±3.5)
**Physical activity** (MET-hours/week) † [39]	34.7 (15.8-64.5)	39.3 (20.4-80.3)	36.5 (13.1-75.0)	43.6 (16.4-77.0)
**Smoking** ‡ [5]				
Never smoker	562 (27.3%)	8 (38.1%)	38 (27.9%)	57 (35.4%)
Past smoker	952 (46.2%)	8 (38.1%)	66 (48.5%)	78 (48.4%)
Current smoker	548 (26.6%)	5 (23.8%)	32 (23.5%)	26 (16.1%)
**Alcohol** (gram/week) † [42]	48.3 (6.9-121.6)	41.5 (13.2-120.1)	44.0 (4.2-115.6)	33.3 (6.9-111.2)
**Diabetes mellitus** ‡	416 (20.2%)	4 (19.0%)	29 (21.5%)	23 (14.2%)
**Total cholesterol** (mg/dl)* [8]	224.2 (±38.2)	229.1 (±31.7)	228.6 (±38.1)	228.1 (±40.4)
**LDL-cholesterol** (mg/dl)* [16]	144.7 (±35.7)	151.3 (±28.9)	148.8 (±33.3)	146.6 (±34.9)
**HDL-cholesterol** (mg/dl)* [10]	51.0 (±14.5)	53.6 (±11.4)	51.4 (±14.1)	50.1 (±13.1)
**Statin intake** ‡ [171]	247 (12.9%)	2 (10.5%)	15 (11.7%)	25 (16.6%)
**High milk consumption** ‡ [126]	571 (29.2%)	9 (45.0%)	32 (25.0%)	43 (28.1%)
**High yoghurt/quark consumption** ‡ [104]	839 (42.5%)	7 (33.3%)	59 (45.5%)	78 (50.6%)
**High cheese consumption** ‡ [84]	980 (49.1%)	11 (55.0%)	86 (51.1%)	81 (52.9%)
**High fruits consumption** ‡ [41]	1,226 (60.5%)	15 (71.4%)	87 (64.0%)	100 (62.9%)
**High vegetable consumption** ‡				
Raw [42]	504 (24.9%)	8 (38.1%)	33 (24.3%)	43 (27.0%)
Cooked [40]	599 (29.5%)	10 (47.6%)	38 (27.9%)	58 (36.5%)
**High fish consumption** ‡ [43]	696 (34.4%)	13 (61.9%)	45 (33.1%)	66 (41.5%)
**PCa in first degree relevants (familiy history)**	59 (2.9%)	1 (5.3%)	4 (2.9%)	3 (2.4%)

* mean (± standard deviation), ‡ number (%), † median (inter quartile range), [number of missings].

Table S-2: Complete case sensitivity analysis (N=1,938): Hazard ratios (HR) and corresponding 95%-confidence intervals (95%-CI) for the association of monoclonal gammopathy of undetermined significance (MGUS) and prostate cancer (PCa), subsequently adjusted for potential risk factors.

Model	HR	95%-CI
**Basic model (BM)**	1.99	1.15-3.43
**BM + BMI**	1.97	1.14-3.40
**BM + physical activity**	1.99	1.15-3.44
**BM + smoking**	2.00	1.16-3.46
**BM + alcohol consumption**	1.99	1.15-3.44
**BM + diabetes mellitus**	1.94	1.12-3.36
**BM + total cholesterol**	1.92	1.11-3.33
**BM + LDL-cholesterol**	1.96	1.13-3.38
**BM + HDL-cholesterol**	2.00	1.16-3.45
**BM + intake of statins**	2.01	1.16-3.47
**BM + milk consumption**	1.99	1.15-3.44
**BM + yoghurt/quark consumption**	1.98	1.15-3.43
**BM + cheese consumption**	1.99	1.15-3.43
**BM + fruits consumption**	2.01	1.16-3.47
**BM + vegetables consumption (raw)**	1.98	1.15-3.42
**BM + vegetables consumption (cooked)**	1.99	1.15-3.44
**BM + fish consumption**	1.99	1.15-3.43
**BM + family history PCa**	1.98	1.15-3.43
**Full Model**	2.03	1.17-3.53

Basic model = adjusted for age and education, BMI = body mass index, LDL = low density lipoprotein, HDL = high density lipoprotein, Full model = adjusted for age, education and all potential risk factors except total cholesterol.

Table S-3: Odds ratios (OR) and corresponding 95%-confidence intervals (95%-CI) for the association of potential risk factors and monoclonal gammopathy of undetermined significance (MGUS) adjusted for age and education.

**Potential risk factor**	**N**	**N_{cases}**	**OR**	**95%-CI**
**Age**	2,375	156	1.04	1.02-1.07
**Education**	2,375	156		
≤ 10 years	120	5	0.39	0.15-1.03
11-13 years	1,135	65	0.55	0.34-0.87
14-17 years	796	57	0.70	0.44-1.12
≥ 18 years	324	29	Ref.	
**BMI** (kg/m^2)^A	2,365	155	1.17	1.00-1.37
**Physical activity** (MET-h/week)^A	2,336	151	0.96	0.81-1.14
**Smoking**	2,375	156		
Never smoker	665	46	Ref.	
Past smoker	1,101	86	0.95	0.65-1.40
Current smoker	609	31	1.07	0.68-1.69
**Alcohol** (g/week)^A	2,333	153	1.03	0.88-1.22
**Diabetes mellitus**	2,375	156	1.02	0.68-1.52
**Total cholesterol** (g/dl)^A	2,367	156	1.13	0.97-1.33
**LDL-cholesterol** (g/dl)^A	2,359	155	1.15	0.98-1.35
**HDL-cholesterol** (g/dl)^A	2,365	156	1.03	0.88-1.21
**Statin intake**	2,208	146	0.75	0.44-1.27
**High milk consumption**	2,252	147	0.85	0.58-1.24
**High yoghurt-/quark consumption**	2,274	150	0.93	0.67-1.31
**High cheese consumption**	2,295	151	1.08	0.77-1.51
**High fruits consumption**	2,337	156	1.05	0.74-1.48
**High vegetables consumption (raw)**	2,336	156	0.99	0.68-1.43
**High vegetables consumption (cooked)**	2,338	156	0.87	0.61-1.25
**High fish consumption**	2,335	156	0.97	0.69-1.37
**Family history PCa**	2,375	156	1.18	0.46-2.99

^{A} = OR per standard deviation (STD) (STD_{BMI}=3.95 kg/m^2, $STD_{physical\_activity.}$=49.58 MET-h/week, STD_{Alc} =129.50 g/week, $STD_{total\_cholesterol.}$=38.27 g/dl, STD_{LDL}=35.49 g/dl, STD_{HDL}=14.35 g/dl), Ref. = reference group.

Table S-4: Hazard ratios (HR) and corresponding 95%-confidence intervals (95%-CI) for the association of potential risk factors and prostate cancer (PCa).

**Potential risk factor**	**N**	$\mathbf{N_{cases}}$	**HR**	**95%-CI**
**Age**	2,346	143	1.05	1.03-1.08
**Education**	2,336	143		
≤ 10 years	119	7	1.13	0.47-2.76
11-13 years	1116	65	1,11	0.64-1.92
14-17 years	782	55	1.29	0.74-2.25
≥ 18 years	319	16	Ref.	
**BMI** (kg/m^2) ^{A}	2,326	143	1.07	0.91-1.25
**Physical activity** (MET-h/week) ^{A}	2,298	143	1.02	0.88-1.20
**Smoking**	2,336	143		
Never smoker	651	51	Ref.	
Past smoker	1,082	67	0.76	0.52-1.09
Current smoker	603	25	0,65	0.40-1.06
**Alcohol** (g/week)^A	2,299	143	0.89	0.74-1.08
**Diabetes mellitus**	2,336	143	0.53	0.32-0.88
**Total cholesterol** (g/dl) ^{A}	2,328	142	1.12	0.95-1.32
**LDL-cholesterol** (g/dl) ^{A}	2,320	142	1.08	0.92-1.27
**HDL-cholesterol** (g/dl) ^{A}	2,327	142	0.93	0.79-1.11
**Statin intake**	2,171	133	1.08	0.67-1.74
**High milk consumption**	2,216	137	1.06	0.74-1.52
**High yoghurt-/quark consumption**	2,239	140	1.08	0.77-1.51
**High cheeseconsumption**	2,259	137	1.07	0.76-1.51
**High fruits consumption**	2,300	143	0.81	0.58-1.14
**High vegetables consumption (raw)**	2,299	143	1.05	0.72-1.53
**High vegetables consumption (cooked)**	2,301	143	1.24	0.88-1.75
**High fish consumption**	2,298	143	1.29	0.92-1.81
**Family history PCa**	2,336	143	0.98	0.36-2.65

^{A} = per standard deviation (STD) (STD_{BMI}=3.97 kg/m^2, $STD_{physical\ activity.}$=48.92 MET-h/week, STD_{Alc} =129.97 g/week, $STD_{total\_cholesterol.}$=38.31 g/dl, STD_{LDL}=35.59 g/dl, STD_{HDL}=14.36 g/dl), Ref. = reference group.
